# Disentangling the influence of entanglement on recruitment in North Atlantic right whales

**DOI:** 10.1098/rspb.2024.0314

**Published:** 2024-03-13

**Authors:** Joshua Reed, Leslie New, Peter Corkeron, Robert Harcourt

**Affiliations:** ^1^ School of Natural Sciences, Macquarie University, North Ryde, New South Wales, 2109, Australia; ^2^ Department of Mathematics, Computer Science and Statistics, Ursinus College, Collegeville, PA, 19426, USA; ^3^ Centre for Planetary Health and Food Security, Griffith University, Nathan, Queensland 4111, Australia

**Keywords:** multi-event mark-recapture, Bayesian, entanglement severity, endangered species

## Abstract

North Atlantic right whales are Critically Endangered and declining, with entanglements in fishing gear a key contributor to their decline. Entanglement events can result in lethal and sub-lethal (i.e. increased energetic demands and reduced foraging ability) impacts, with the latter influencing critical life-history states, such as reproduction. Using a multi-event framework, we developed a Bayesian mark-recapture model to investigate the influence of entanglement severity on survival and recruitment for female right whales. We used information from 199 known-aged females sighted between 1977 and 2018, combined with known entanglements of varying severity that were classified as minor, moderate or severe. Severe entanglements resulted in an average decline in survival of 27% for experienced non-breeders, 9% for breeders and 26% for pre-breeding females compared with other entanglements and unentangled individuals. Surviving individuals with severe entanglements had low transitional probabilities to breeders, but surprisingly, individuals with minor entanglements had the lowest transitional probabilities, contrary to expectations underpinning current management actions. Management actions are needed to address the lethal and sub-lethal impacts of entanglements, regardless of severity classification.

## 1. Introduction

North Atlantic right whales are currently listed as Critically Endangered on the IUCN Red List and are the second most imperilled great whale species, with fewer than 350 individuals alive [1–4]. Since 2014, the number of female North Atlantic right whales has steadily declined, with only an estimated 142 (95%CI: (135,150)) remaining at the start of 2018 [5]. However, the overall decline has been uneven, with the abundance of reproductively mature females declining post-2014 to an estimated 73 individuals, or 51% of the female population, whereas the abundance of pre-breeders plateaued between 2010 and 2018 at an estimated 68 individuals [5]. This failure of female North Atlantic right whales to recruit to the breeding population is considered a key driver for the decline in recent years in reproductively mature females, and therefore the entire species [5]. Individual female North Atlantic right whales can mature as young as 5 years old, although historically the average age of first parturition was estimated to be 9 years [6]. Despite intensive study, many individuals much older than 9 years have no confirmed reproductive events [2]. Poor body condition as a result of anthropogenic and environmental stressors could significantly influence female North Atlantic right whale recruitment into the breeding population [7]. In addition, recent work has found that maternal body length has decreased over time, also reducing fecundity, given that larger individuals have shorter interbirth intervals, produce more calves per reproductively available year and have higher lifetime reproductive output compared with smaller whales [8].

Between 1990 and 2017, 41 non-calf North Atlantic right whale carcasses were recovered, with 21 linked to vessel collisions and 20 to entanglements. In the same period, 62 serious injuries were recorded, with eight resulting from vessel collisions and 54 from entanglements [9], highlighting entanglements as a leading cause of mortality. North Atlantic right whales are protected under the Endangered Species Act and Marine Mammal Protection Act (MMPA) in the USA, and the Species at Risk Act in Canada, both with the stated aim of ensuring the recovery of this species by reducing anthropogenic threats. Since 1997, the US National Marine Fisheries Service has implemented a number of regulations intended to reduce the severity and frequency of entanglement events. However, over the last decade, instances of moderate and severe entanglements have increased, and these negatively impact health and survival rates, particularly for reproductive females [10–12]. Deleterious consequences of entanglement in fishing gear, such as increased drag and reduced foraging ability [13,14] increase with entanglement severity, and can lead to affected whales entering a state of reduced energy balance, impacting their ability to recover from these events [15]. Since 2017, an Unusual Mortality Event has been declared for the North Atlantic right whale, triggering a ‘serious injury determination assessment’ as required under the MMPA, wherein injuries are designated as serious (indicating the individual is likely to die from its injuries), or non-serious [16]. Currently, the majority of entanglement events in North Atlantic right whales are considered ‘non-serious’ and are recorded as ‘minor’.

For right whales, entanglements can be as energetically demanding as migratory or reproductive events [17]. However, unlike energetic demands based on the species' life history, entanglement events occur at random time points relative to an individual's reproductive or migratory cycle. It is a matter of chance whether an individual has sufficient reserves to endure the energetic costs of entanglement at the time it encounters fishing gear. This can result in biologically significant consequences. For instance, recent research on the sub-lethal effects of entanglement, ship strikes and climate-driven changes in prey availability, has shown that North Atlantic right whales who have been entangled with attached gear, or whose mother was entangled during nursing, are on average 0.64 and 0.69 m shorter, respectively, than their expected age-specific length, based upon species-specific growth curves [18]. This induced stunting affects the individuals' ability to accumulate energy reserves, which impacts calf production [8]. Prior work has demonstrated that more than 80% of the species has experienced at least one entanglement event, with nearly 60% being entangled twice or more in their lifetime [19]. Given the frequency of entanglement, understanding its influence on reproduction is important if attempting to forecast the population dynamics of this species.

For populations of free-ranging mammals, determining an individual's reproductive history remains challenging due to the uncertainty in assigning reproductive state and the imperfect detections of individuals [20–22]. Modified multi-state mark-recapture models have been developed to account for state uncertainty by introducing the concept of events [23,24], or the underlying, but hidden, states of interest. The North Atlantic right whale occurs along the east coast of North America, with a well-documented seasonal migration between calving areas off the coast of the southeastern USA and feeding grounds off the northeastern coast of USA and Canada [25–27]. Migratory events are not always undertaken by all individuals within the population, with individuals often detected outside the locations in which North Atlantic right whales seasonally aggregate [6,25,27,28]. Reproductive events in North Atlantic right whales may be missed due to calves being lost before being recorded, or reproduction occurring outside monitored areas. Currently, most known aged individuals enter the catalogue at six months [2], thus multi-state mark-recapture models can help address the issue of imperfect observations.

Using a multi-state framework, we fit a Bayesian mark-recapture model accounting for uncertainty in the assignment of reproductive states to assess the influence of (i) entanglement severity in fishing gear, and (ii) the number of entanglements previously experienced by an individual, on the recruitment of individuals into the breeding population for female North Atlantic right whales. We also assessed the state-specific apparent survival and recapture probabilities for female North Atlantic right whales. We use this information to make inferences on the drivers for the poor species recovery and recent decline in abundance.

## 2. Methods

A total of 41 years of data from 1977 to 2018, for 199 known age North Atlantic right whales were used, with surveys of varying intensity conducted yearly [2,29].

### (a) Covariates

**Figure 1.**
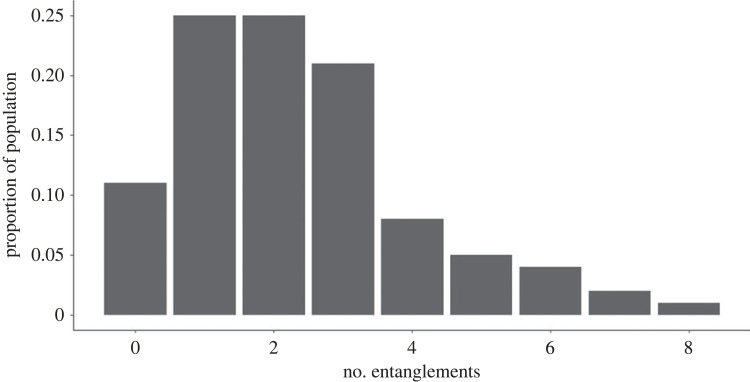
Information on the number of entanglements experienced by female North Atlantic right whales.

Information on an individual's entanglement score and birth cohort was compiled from the North Atlantic Right Whale Consortium sighting, identification [2] and entanglement [30] databases. Using the entanglements database, we calculated the number of entanglements previously experienced by an individual and grouped them based on the number of entanglements. Individuals with four or more entanglements were combined into one group due to the small sample size (figure 1). We used entanglement injury severity level to assign entanglement score, as defined by the North Atlantic Right Whale Consortium [30], where individuals were assigned to one of four states: (1) not entangled, (2) minor entanglement, (3) moderate entanglement and (4) severe entanglement. Entanglement score was assigned as an individual, time-varying covariate, where only the entanglement score observed in a given year was used to define the covariates' value. These scores were derived from the Right Whale Consortium, with a description of how the scores are determined found in the electronic supplementary material (*Entanglement severity*). They are similar to the ‘determinations’ made by National Oceanic and Atmospheric Administration (NOAA) under the MMPA, but slightly different, in that they focus on the severity of damage caused to the individual by the entanglement, rather than a ‘determination’ based upon an assessment of whether an individual is likely to survive the entanglement event.

### (b) Analysis

A multi-state mark-recapture model was created to assess the uncertainty in breeding state assignment and reproductive histories. We considered four true biological states which correspond to four stages of the species life history: pre-breeders (PB), prior to an individual starting to calve; breeders (B), in the year an individual calved; experienced non-breeders (ENB), mature females between calving years; and dead (D). Five possible observations could be made from the data: (1) seen and assigned as a pre-breeder, (2) seen with unknown breeding state, (3) seen and assigned as a breeder, (4) seen and assigned as an experienced non-breeder (a non-reproductive state of females who have previously calved), and (5) not seen. If an individual was seen with a calf, they were assigned as a breeder, if an individual was younger than 5 years they were considered pre-breeders, until they had successfully brought a calf to term. Due to the high sampling effort for North Atlantic right whales, we only assigned an unknown breeding state to individuals who had at least one sighting gap between 5 years of age and first recorded calving event. All individuals were first sighted as pre-breeders (sighted in year of birth).

**Figure 2.**
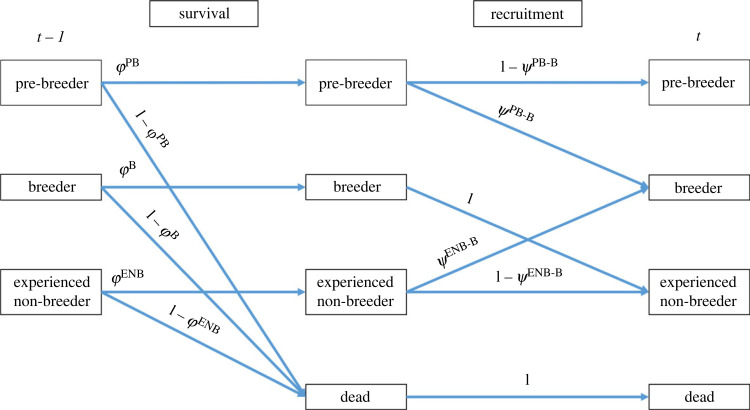
Graphical depiction of the state model, with movement between states displayed by arrows. Model notation: arrows represent survival (*φ*) and recruitment (*ψ*), with superscripts referring to the state of the individual, being pre-breeder (PB), breeder (*b*) and experienced non-breeder (ENB).

Our model included four different parameters: survival probability (*φ*), the probability of recruitment (transitioning between non-reproductive states to breeder) (*ψ*), recapture probability (*p*) and state assignment probability (*δ*). Recapture, recruitment and survival probability were modelled as

logit( p(i,t))=Ent(i,t)+ε(t),

logit(ψ(i,t))=Ent(i,t)+N.Ent(i,t)

andlogit(φ(i,t))=Ent(i,t)+N.Ent(i,t),

where Ent is the additive effect of the entanglement covariate for individual *i* at time *t*, *ε* is the random effect of time *t*, N.Ent is the additive effect of the number of entanglements experienced by individual *i* at time *t*. A multinomial tree diagram is presented showing a visual representation of the model (figure 2).

The state process can be represented by a transition matrix with the individual's state at time *t* represented by the rows, and the state at *t +* 1 represented by the columns,

$$\begin{array}{rl} & \;\;\;\;\;\;\mathrm{PB}\;\;\;\;\;\;\;\;\;\;\;\;\;\;\;\;\;\;\;\;\;\;\;\;B\;\;\;\;\;\;\;\;\;\;\;\;\;\;\;\;\;\;\;\mathrm{ENB}\;\;\;\;\;\;\;\;\;\;\;\;\;\;\;\;\;\;\;\;\;\;\;\;\;\;\mathrm{Dead}\\ & \begin{array}{c} \mathrm{PB}\\ B\\ \mathrm{ENB}\\ \mathrm{Dead} \end{array}\left[\begin{array}{c} \varphi^{\mathrm{PB}}(1-\psi^{\mathrm{PB}-B})\\ 0\\ 0\\ 0 \end{array}\;\begin{array}{c} \varphi^{\mathrm{PB}}(1-\psi^{\mathrm{PB}-B})\;\\ 0\\ \varphi^{\mathrm{ENB}}\psi^{\mathrm{ENB}-B}\\ 0 \end{array}\begin{array}{c} 0\\ \varphi^{B}\\ \varphi^{ENB}(1-\psi^{\mathrm{ENB}-B})\\ 0 \end{array}\;\;\;\;\begin{array}{c} 1-\psi^{\mathrm{PB}}\\ 1-\varphi^{B}\\ 1-\varphi^{\mathrm{ENB}}\\ 1 \end{array}\right]. \end{array}$$

The observation process can be represented by a row stochastic matrix, with rows representing the states and the columns representing the observations (1: seen and assigned as a pre-breeder, 2: seen with unknown breeding state, 3: seen and assigned as a breeder, 4: seen and assigned as an experienced non-breeder and 5: not seen),

$$\begin{array}{rl} & \;\;\;\;\;1\;\;\;\;\;\;\;\;\;\;\;\;\;\;\;\;2\;\;\;\;\;\;\;\;\;\;\;\;\;\;\;3\;\;\;\;\;\;\;\;\;\;\;\;\;\;\;\;\;\;\;\;\;\;\;\;\;\;\;\;\;\;\;\;\;\;\;\;4\;\;\;\;\;\;\;\;\;\;\;\;\;\;\;\;\;\;\;\;\;\;\;\;\;\;5\\ & \begin{array}{c} \mathrm{PB}\\ B\\ \mathrm{ENB}\\ \mathrm{Dead} \end{array}\left[\begin{array}{c} p^{\mathrm{PB}}(1-\delta^{\mathrm{PB}})\\ 0\\ 0\\ 0 \end{array}\;\begin{array}{c} p^{\mathrm{PB}}\delta^{\mathrm{PB}}\;\\ p^{B}\delta^{B}\\ p^{\mathrm{ENB}}\delta^{\mathrm{ENB}}\\ 0 \end{array}\;\begin{array}{c} 0\\ p^{B}(1-\delta^{B})\\ 0\\ 0 \end{array}\;\;\;\;\begin{array}{c} 0\\ 0\\ p^{\mathrm{ENB}}(1-\delta^{\mathrm{ENB}})\\ 0 \end{array}\;\begin{array}{c} 1-p^{\mathrm{PB}}\\ 1-p^{B}\\ 1-p^{\mathrm{ENB}}\\ 1 \end{array}\right]. \end{array}$$

Uniform priors, *U*(0, 1), were assigned to all modelled parameters, except for the random covariate of entanglement history and cohort, which were given by *U*(−10, 10) on the logit scale. The general structure of the joint posterior for this multi-event model is provided in the electronic supplementary material (*Model Description*).

Data were modelled using the program Stan (v. 2.28) [31,32] via R (v. 4.1.1) [33] using the package ‘Rstan’ (v. 2.21.2) [34]. We performed 3000 iterations with a burn-in phase of 1500 iterations, run over three chains with no thinning. The model took 2 h to fit, running on a desktop with an i5 processor and 16GB RAM. Convergence was assessed both visually and using the R-hat statistic, were an R-hat value of 1.1 or less indicating convergence. Stan code and model output can be found in the electronic supplementary material (*Code*, table S1).

## 3. Results

Information on 199 known aged female right whales was used for this study. Individual entanglement histories revealed that 89% of these individuals had experienced at least one entanglement event in their life, with 49 of those individuals experiencing two recorded entanglement events, 42 individuals experiencing three entanglements, and 37 having four or more entanglements, with one individual suffering from eight entanglement events (figure 1). Only 22 individuals (11%) had never been entangled at all throughout their life. The mean age of last sighting for this small subset of the population is 2.1 years (range: 0–8). A breakdown of entanglement severity revealed that at least once in their life 164 individuals experienced minor entanglements, 63 experienced moderate entanglements and 24 experienced a severe entanglement.

**Figure 3.**
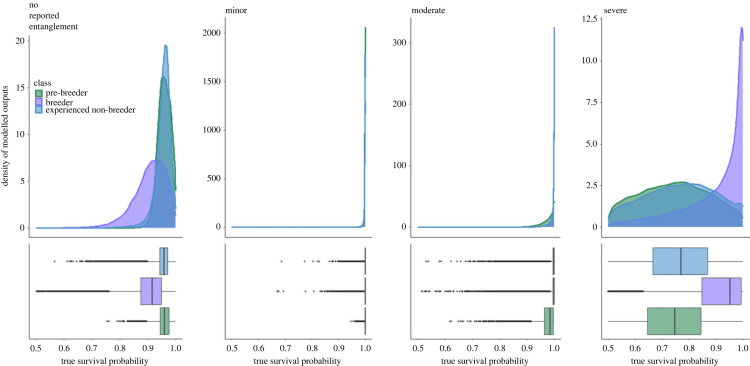
Apparent survival estimates for female North Atlantic right whales. Posterior distributions for apparent survival by entanglement severity score and class (pre-breeder (green), breeder (purple) and experienced non-breeder (blue)). Box plots show the mean and spread of the estimates.

Mean apparent survival estimates for pre-breeders were highest for individuals who experienced a minor entanglement in a given year at 0.999 (95%CI: (0.989,1)) (figure 3 and table 1), while individual pre-breeders with no reported entanglements in a given year also showed high apparent survival at 0.959 (95%CI: (0.899,0.999)). Pre-breeders with moderate entanglements had a mean apparent survival estimate of 0.9975 (95%CI: (0.875,1)), while individuals who experienced a severe entanglement in a given year showed the lowest apparent survival estimates, at 0.707 (95%CI: (0.317,0.995)) (figure 3 and table 1).

**Table 1.** Average mean yearly apparent survival scores and 95% credible intervals (CI) for individual classes and birth cohorts (before 2000, after 2000), for each yearly entanglement score. Mean values are the average across the number of past entanglements an individual has experiences.

entanglement score	no entanglement		minor		moderate		severe	
class	mean	95%CI range	mean	95%CI range	mean	95%CI range	mean	95%CI range
pre-breeder	0.959	0.899–0.999	0.999	0.980–1.000	0.975	0.867–1.000	0.707	0.317–0.995
breeder	0.906	0.655–0.997	0.998	0.978–1.000	0.991	0.914–1.000	0.882	0.371–1.000
experienced non-breeder	0.954	0.798–0.999	0.998	0.981–1.000	0.992	0.913–1.000	0.724	0.212–0.998

Breeders with minor entanglements showed the highest apparent survival rates, with a yearly average of 0.998 (95%CI: (0.978,1)), while individuals who had not been recorded entangled had an average annual apparent survival probability of 0.906 (95%CI: (0.655,0.997)) for breeders (figure 3 and table 1). Apparent survival for breeders with moderate entanglements had an annual average of 0.991 (95%CI: (0.914,1)), while female breeders with severe entanglements had the lowest apparent survival with an annual average of 0.882 (95%CI: (0.371,1)) (figure 3 and table 1).

Apparent survival per year for experienced non-breeders was highest for individuals with minor entanglements at 0.998 (95%CI: (0.981,1)), while experienced non-breeders with no recorded entanglement events in a given year had an average annual apparent survival rate of 0.954 (95%CI: (0.798,0.999)) (figure 3 and table 1). Individuals with moderate entanglements had an average apparent survival of 0.992 (95%CI: (0.913,1)), while experienced non-breeders with severe entanglements had the lowest apparent survival rates at 0.724 (95%CI: (0.212,0.998)) (figure 3 and table 1). A full breakdown of the influence of the number of previous entanglements on apparent survival can be found in the electronic supplementary material, figures S1–S3 and table S1.

**Figure 4.**
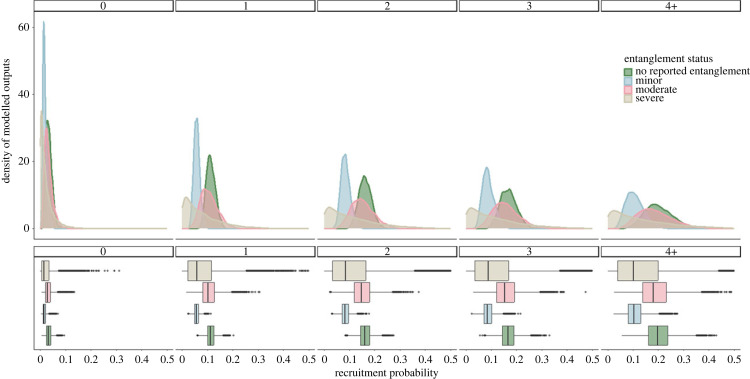
Recruitment probability estimates for female North Atlantic right whales. Posterior distributions for yearly recruitment by entanglement severity score (no reported entanglement (green), minor (blue), moderate (pink) and severe (beige)). Box plots show the mean and spread of the estimates.

Recruitment of individuals into the breeding population from pre-breeders was influenced by entanglement status and number of previous entanglements. Individuals with no recorded entanglements in a given year had a mean annual recruitment probability of 0.033 (95%CI: (0.013,0.061)) with zero past entanglements, 0.113 (95%CI: (0.081,0.152)) with one, 0.163 (95%CI: (0.115,0.216)) with two, 0.169 (95%CI: (0.108,0.240)) with three and 0.201 (95%CI: (0.105,0.321)) with four plus past entanglements (figure 4 and table 2). Individuals with minor entanglements had the lowest transition probability at 0.017 (95%CI: (0.006,0.036)) with zero past entanglements, 0.057 (95%CI: (0.036,0.083)) with one, 0.085 (95%CI: (0.053,0.125)) with two, 0.089 (95%CI: (0.050,0.143)) with three and 0.108 (95%CI: (0.049,0.193)) with four plus past entanglements (figure 4 and table 2). Females with moderate entanglements had a mean transition probability of 0.032 (95%CI: (0.009,0.073)) for individuals with zero previous entanglements, 0.107 (95%CI: (0.050,0.180)) with one, 0.153 (95%CI: (0.077,0.312)) with two, 0.159 (95%CI: (0.072,0.274)) with three and 0.189 (95%CI: (0.079,0.274)) with four plus entanglements; and for individuals with severe entanglements 0.025 (95%CI: (0.000,0.106)) with zero past entanglements, 0.080 (95%CI: (0.001,0.284)) with one, 0.114 (95%CI: (0.001,0.370)) with two, 0.118 (95%CI: (0.001–0.390)) with three and 0.140 (95%CI: (0.001,0.464)) with four plus past entanglements (figure 4 and table 2).

**Table 2.** Yearly mean recruitment probabilities and 95% credible intervals (CI) for female right whales in two birth cohorts (before 2000, after 2000) and number of previous entanglements experienced, for each yearly entanglement score.

entanglement score	no entanglement		minor		moderate		severe	
number of entanglements	mean	95%CI	mean	95%CI	mean	95%CI	mean	95%CI
0	0.033	0.013–0.061	0.017	0.006–0.036	0.032	0.009–0.073	0.025	0.000–0.106
1	0.113	0.081–0.152	0.057	0.036–0.083	0.107	0.050–0.184	0.080	0.001–0.284
2	0.163	0.115–0.216	0.085	0.053–0.125	0.153	0.077–0.251	0.113	0.001–0.370
3	0.169	0.108–0.240	0.089	0.050–0.143	0.159	0.072–0.274	0.118	0.001–0.390
4+	0.201	0.105–0.321	0.108	0.049–0.193	0.189	0.079–0.342	0.140	0.001–0.464

**Figure 5.**
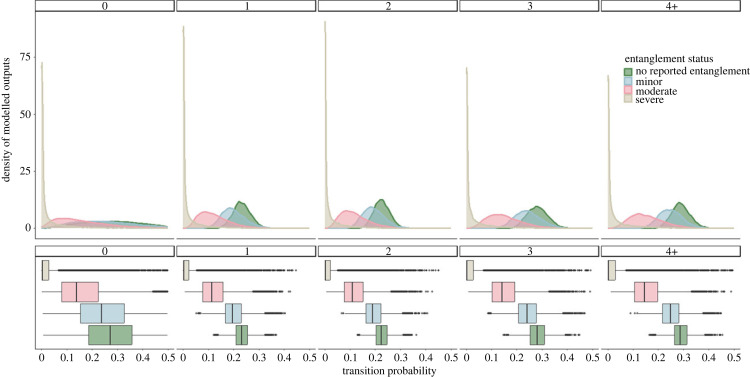
Transition probability estimates for female North Atlantic right whales. Posterior distributions for yearly transition probability from experienced non-breeder to breeder by entanglement severity score (no reported entanglement (green), minor (blue), moderate (pink) and severe (beige)). Box plots show the mean and spread of the estimates.

Transition probability from experienced non-breeder to breeder was the lowest for individuals with severe entanglements regardless of the number of entanglements, with a mean transition probability of 0.036 (95%CI: (0.000,0.296)) with zero, 0.026 (95%CI: (0.000,0.199)) with one, 0.025 (95%CI: (0.000,0.193)) with two, 0.032 (95%CI: (0.000,0.241)) with three and 0.033 (95%CI: (0.000, 0.241)) with four plus past entanglements (figure 5 and table 3). Individuals with moderate entanglements were also seen to have low transition probability at 0.171 (95%CI: (0.022, 0.486)) for individuals with zero previous entanglements, 0.123 (95%CI: (0.036, 0.253)) with one, 0.119 (95%CI: (0.036, 0.253)) with two, 0.152 (95%CI: (0.046, 0.311)) with three and 0.155 (95%CI: (0.049, 0.304)) with four plus past entanglements (figure 5 and table 3). Minor entanglements resulted in a transition probability of 0.264 (95%CI: (0.054, 0.570)) for females with zero past entanglements, 0.200 (95%CI: (0.117, 0.307)) with one, 0.194 (95%CI: (0.119, 0.292)) with two, 0.244 (95%CI: (0.149, 0.354)) with three and 0.250 (95%CI: (0.165, 0.353)) with four plus past entanglements (figure 5 and table 3). Individuals with no recorded entanglements had the highest transition probabilities, with a mean of 0.300 (95%CI: (0.069,0.606)) with zero past entanglements, 0.232 (95%CI: (0.165, 0.303)) with one, 0.226 (95%CI: (0.167, 0.292)) with two, 0.282 (95%CI: (0.205, 0.362)) with three and 0.289 (95%CI: (0.219, 0.366)) with four plus past entanglements (figure 5 and table 3).

**Table 3.** Yearly mean transition probabilities and 95% credible intervals (CI) for female right whales in two birth cohorts (before 2000, after 2000) and number of previous entanglements experienced, for each yearly entanglement score.

entanglement score	no entanglement		minor		moderate		severe	
number of entanglements	mean	95%CI	mean	95%CI	mean	95%CI	mean	95%CI
0	0.300	0.069–0.606	0.264	0.054–0.570	0.171	0.022–0.486	0.036	0.000–0.296
1	0.232	0.165–0.303	0.200	0.117–0.307	0.123	0.036–0.273	0.026	0.000–0.199
2	0.226	0.167–0.292	0.194	0.119–0.292	0.119	0.036–0.253	0.025	0.000–0.193
3	0.282	0.205–0.362	0.244	0.149–0.354	0.152	0.046–0.311	0.032	0.000–0.241
4+	0.289	0.219–0.366	0.250	0.165–0.353	0.155	0.049–0.304	0.033	0.000–0.241

Recapture probability between years remained consistent for pre-breeders, regardless of entanglement status (electronic supplementary material, figure S4). Breeding females showed the lowest overall recapture probability across the study period, with notable declines prior to 2000 and after 2015 (electronic supplementary material, figure S4). Recapture probabilities for experienced non-breeders remained constant in trend regardless of entanglement status. Individuals with minor entanglements saw the lowest recapture probabilities (electronic supplementary material, figure S4).

Unknown state assignment, or the probability of correctly assigning an individual to a given state, was lowest for experienced non-breeders with a mean probability of 0.634 (95%CI: (0.600,0.667)) of correctly assigning individuals, while the unknown state assignment probability for pre-breeders was 0.999 (95%CI: (0.996,1)), and 0.978 (95%CI: (0.927,0.999)) for breeders, with the inverse of these probabilities being the probability that an individual in an unknown state would be incorrectly assigned to that state.

## 4. Discussion

Entanglement in fishing gear is one of the key threats facing North Atlantic right whales. Almost all females, 89%, have experienced at least one entanglement event during their lifetime, and almost two-thirds of these have experienced two or more, with many resulting in injury or mortality. Consistent with previous work [19,35], we found that female North Atlantic right whales who experience a severe entanglement have reduced survival probability. Pre-breeders subject to at least one severe entanglement show an average reduction in survival of 27% compared with pre-breeders with moderate, minor or no entanglements. Breeding females subject to one severe entanglement had a mean reduction in apparent survival of 9% compared with other entanglement scores, while experienced non-breeders had an average reduction in apparent survival of 26% after experiencing a severe entanglement, compared with other entanglement scores (minor, moderate, severe, not recorded entangled) (figure 3). This is consistent with earlier work reporting that severely entangled individuals had a mean apparent survival probability of 0.562 in the immediate sighting period after the entanglement, and 0.738 for subsequent sightings [36].

Results from our model also demonstrate that females with no reported entanglements had reduced apparent survival probabilities when compared with individuals with minor or moderate entanglement severity scores. This was particularly the case for breeding females (figure 3). An important caveat to consider when interpreting these results is that the model uses the entanglement severity score that the individual was assigned in each annual time step. This means an individual could have suffered an entanglement event and been disentangled or shed the rope and/or gear, resulting in it being not entangled in the following year when it died, for example catalogue no. Eg 2301, who was severely entangled from September 2003 until September 2004, dying 178 days after the end of the last reported entanglement event in March 2005. These deaths could have been a result of complications or infections from a past entanglement, mortality from another source, i.e. vessel strike, or even as a result of an unobserved entanglement, with observed carcasses only accounting for 36% of the estimated mortality of North Atlantic right whales [9].

Our model shows that pre-breeders who survived while experiencing a minor or severe entanglement in a particular year were less likely to transition into the breeding population compared with those who were not entangled or who had moderate entanglements. Surprisingly, those with minor entanglements showed the lowest recruitment probabilities out of all entanglement categories, with individuals being on average 47% less likely to transition compared with non-entangled whales (figure 4). Individuals with severe entanglements had a lower mean recruitment probability than non-entangled individuals, though there was a greater level of uncertainty in estimates for severe entanglements (figure 4). Within the model framework we allowed for the fact that females needed to be at least 5 years old before they could recruit. We recognize this could influence the results, due to the inherent low number of individuals who had never experienced an entanglement event in their lifetime.

Individuals who had not experienced any previous entanglements showed the lowest recruitment probabilities (figure 4). Given the prevalence of entanglement in the population, this is probably a result of this group being composed of mostly young individuals who have not yet reached minimum age of first parturition (5 years). The small number of individuals (*n* = 22) with a mean age of 2 (range: 0–8) had never been recorded with an entanglement in their lifetime. For individuals over the age of 5 before experiencing their first entanglement, the oldest known aged female was 16, with a mean age of 7 (range: 5–16). Recruitment estimates were highly variable, particularly for individuals with moderate and severe entanglements compared with those with minor entanglements. This variability is probably the result of two factors: (i) the small sample size for individuals experiencing moderate (63 individuals) and severe entanglements (24 individuals) compared with other entanglement scores (164 individuals with minor); and (ii) that recruitment is conditional on survival. The small sample size explains the variability in the estimates of recruitment for moderately and severely entangled individuals, and the apparent high probability of recruitment associated with these entanglements is explained by the conditional nature of recruitment. Those individuals that die also fail to recruit, but the loss of those individuals is reflected in the reduced estimate of apparent survival. Therefore, given that an individual is in good enough condition to survive a moderate or severe entanglement, their probability of being able to recruit to the breeding cohort is relatively high. However, many of the individuals who experience moderate or severe entanglement do not survive, so the absolute number of individuals with moderate or severe entanglements to recruit is low.

It has been apparent for some time that North Atlantic right whales with moderate or severe entanglements have reduced immediate apparent survival compared with individuals with minor entanglements [36]. The sub-lethal effects of severe entanglements and gear attachment on North Atlantic right whales was also reasonably well understood, with these individuals more likely to be in poor body condition [15], given that the energetic costs of moderate to severe entanglement is of a similar order of magnitude energetically to reproduction and/or migration [17]. The timing of entanglement events has been shown to influence an individual's capacity to cope with entanglements, with the onset of these events being unpredictable [17], and can affect an individual's specific energetic demands (i.e. lactation, migration) and health (i.e. body condition) at the time of an entanglement, influencing their ability to survive or reproduce as a result of the entanglement [35]. When estimates of recruitment from the model are corrected to account for those individuals who did not survive, using the estimates for apparent survival, we can now estimate ‘true’ recruitment. Estimates of ‘true’ recruitment revealed that for individuals with moderate entanglements, recruitment was approximately 2% lower than estimated and approximately 30% lower for individuals with severe entanglements (table 4).

**Table 4.** List of estimated mean recruitment probabilities for moderate and severe entanglement severity scores compared with their adjusted mean estimates when accounting for apparent survival estimates. Calculated as apparent survival multiplied by the estimated mean recruitment probabilities.

entanglement severity	moderate		severe	
number of entanglements	mean estimate	adjusted	mean estimate	adjusted
0	0.032	0.031	0.025	0.015
1	0.107	0.103	0.080	0.050
2	0.153	0.150	0.113	0.085
3	0.159	0.156	0.118	0.089
4+	0.189	0.186	0.140	0.111

In healthy populations, reproduction in right whales occurs on a 3- to 4-year cycle [37]. In recent years, for North Atlantic right whales the mean inter-calf interval is greater than 7 years [4]. This change in reproductive output has been linked to health, food availability and quality, and anthropogenic pressures [7,12,37]. Our model highlights the influence of entanglement on a female's ability to reproduce after already entering the breeding population. Individuals with severe entanglements have the lowest probability of transitioning to a breeder (figure 5). Individuals with moderate entanglements were 46% on average less likely to transition than individuals with no recorded entanglements. Individuals with minor entanglements were 13% less likely to transition from experienced non-breeder to breeders (figure 5). These findings are supported by recent findings that whales in better health were more likely to successfully calve compared with individuals with entanglements [35].

Abundance of the North Atlantic right whale has been in decline since 2010 [3], with the total number of females declining since 2014. This decline has been driven by anthropogenic mortality of breeding females and a plateau in the number of pre-breeders, resulting in a failure in recruitment to the breeding cohort [5]. North Atlantic right whales face multiple stressors, including vessel collision, entanglement and anthropogenic climate change causing changes to prey quality, abundance and distribution; however, entanglements are the leading cause of mortality for large whales in the western North Atlantic [17], and a leading cause of serious injury for North Atlantic right whales [9]. Currently, management and conservation efforts in the USA for the North Atlantic right whale focus on the reduction of mortality and serious injury from entanglements and vessel strikes [38]. The Department of Fisheries and Oceans Canada have implemented static and dynamic fisheries closures, and increased reporting requirements for lost gear and fishing activities in order to reduce the number of entanglement events occurring within Canadian waters [39]. Our study highlights the significant negative impact of all entanglements on the recruitment probabilities of female North Atlantic right whales, even those entanglements currently considered ‘minor’. We have demonstrated that all entanglements, no matter the severity, can impact the population dynamics of this species through lower recruitment of individuals into the breeding population.

Our analysis indicates that the assumption that ‘minor’ entanglements do not negatively influence the species' population dynamics is incorrect. Entangled individuals are less likely to reproduce, and this decline has been amplified by an overall reduction in reproduction since the turn of the century, possibly as a result of stunting to which entanglement also contributes [8,18]. Management actions that mitigate the occurrence of all entanglements, *regardless of severity*, are needed to reduce both the lethal and sub-lethal effects of entanglement (for example [12,40]). We suggest that only by retargeting management strategies to remove all levels of entanglement will the North Atlantic right whale have the potential to recover. More broadly, we urge caution about dismissing the influence of *any* level of anthropogenic injury on critically endangered species. Further, these findings suggest that entanglements of other marine megafauna may be having greater impacts than currently appreciated, and we encourage other investigators to reconsider whether entanglements classified as ‘minor’ are indeed only having a true minor impact. Finally, we recommend that, given this propensity to mislead, value-laden terms such as ‘minor’, ‘moderate’ and ‘severe’ are no longer used to categorize entanglement injuries and scarring.

## Data Availability

Data are managed and provided by the North Atlantic Right Whale Consortium, requests can be made at: https://www.narwc.org/narwc-databases.html. Supplementary material is available online [41].

## References

[1] Cooke JG. 2020 *Eubalaena glacialis*.*The IUCN Red List of Threatened Species 2020*. (10.2305/IUCN.UK.2020-2.RLTS.T41712A178589687.en)

[2] North Atlantic Right Whale Consortium. 2020 North Atlantic right whale consortium identification and sightings database 01/20/2020. Boston, MA: New England Aquarium.

[3] Pace III RM, Corkeron PJ, Kraus SD. 2017 State–space mark–recapture estimates reveal a recent decline in abundance of North Atlantic right whales. Ecol. Evol. **7**, 8730-8741. (10.1002/ece3.3406) 29152173 PMC5677501

[4] Pettis HM, Pace III RM, Hamilton PK. 2022 North Atlantic Right Whale Consortium 2021 annual report card. In *Report to the North Atlantic Right Whale Consortium*. See https://www.narwc.org/uploads/1/1/6/6/116623219/2021report_cardfinal.pdf.

[5] Reed J, New L, Corkeron P, Harcourt R. 2022 Multi-event modeling of true reproductive states of individual female right whales provides new insights into their decline. Front. Mar. Sci. **9**, 994481. (10.3389/fmars.2022.994481)

[6] Kraus SD, Hamilton PK, Kenney RD, Knowlton AR, Slay CK. 2001 Reproductive parameters of the North Atlantic right whale. J. Cetacean Res. Manage. **2**, 231-236.

[7] Christiansen F et al. 2020 Population comparison of right whale body condition reveals poor state of the North Atlantic right whale. Mar. Ecol. Progress Ser. **640**, 1-16. (10.3354/meps13299)

[8] Stewart JD et al. 2022 Larger females have more calves: influence of maternal body length on fecundity in North Atlantic right whales. Mar. Ecol. Progress Ser. **689**, 179-189. (10.3354/meps14040)

[9] Pace III RM, Williams R, Kraus SD, Knowlton AR, Pettis HM. 2021 Cryptic mortality of North Atlantic right whales. Conserv. Sci. Practice **3**, e346. (10.1111/csp2.346)

[10] Fauquier D et al. 2020 *Report of the Health Assessment Workshop for North Atlantic Right Whales (Eubalaena glacialis), June 24-26, 2019*. NOAA technical memorandum NMFS-OPR 65. (10.25923/jygt-k217)

[11] Knowlton AR, Robbins J, Landry S, McKenna HA, Kraus SD, Werner TB. 2016 Effects of fishing rope strength on the severity of large whale entanglements. Conserv. Biol. **30**, 318-328. (10.1111/cobi.12590) 26183819

[12] Moore MJ et al. 2021 REVIEW assessing North Atlantic right whale health: threats, and development of tools critical for conservation of the species. Dis. Aquat. Organ. **143**, 205-226. (10.3354/dao03578) 33629663

[13] Lambertsen RH, Rasmussen KJ, Lancaster WC, Hintz RJ. 2005 functional morphology of the mouth of the bowhead whale and its implications for conservation. J. Mamm. **86**, 342-352. (10.1644/ber-123.1)

[14] van der Hoop J et al. 2014 Behavioral impacts of disentanglement of a right whale under sedation and the energetic cost of entanglement. Mar. Mamm. Sci. **30**, 282-307. (10.1111/mms.12042)

[15] Pettis HM, Rolland RM, Hamilton PK, Knowlton AR, Burgess EA, Kraus SD. 2017 Body condition changes arising from natural factors and fishing gear entanglements in North Atlantic right whales *Eubalaena glacialis*. Endanger. Species Res. **32**, 237-249. (10.3354/esr00800)

[16] National Marine Fisheries Service (NMFS). 2022 2017–2022 North Atlantic right whale unusual mortality event. See https://www.fisheries.noaa.gov/national/marine-life-distress/2017-2022-north-atlantic-right-whale-unusual-mortality-event.

[17] van der Hoop J, Corkeron P, Moore M. 2017 Entanglement is a costly life-history stage in large whales. Ecol. Evol. **7**, 92-106. (10.1002/ece3.2615) 28070278 PMC5213775

[18] Stewart JD, Durban JW, Knowlton AR, Lynn MS, Fearnbach H, Barbaro J, Perryman WL, Miller CA, Moore MJ. 2021 Decreasing body lengths in North Atlantic right whales. Curr. Biol. **31**, 3174-3179. (10.1016/j.cub.2021.04.067) 34087102

[19] Knowlton AR, Hamilton PK, Marx MK, Pettis HM, Kraus SD. 2012 Monitoring North Atlantic right whale *Eubalaena glacialis* entanglement rates: a 30 yr retrospective. Mar. Ecol. Progress Ser. **466**, 293-302. (10.3354/meps09923)

[20] Buoro M, Prévost E, Gimenez O. 2010 Investigating evolutionary trade-offs in wild populations of Atlantic salmon (*Salmo salar*): incorporating detection probabilities and individual heterogeneity. Evolution **64**, 2629-2642. (10.1111/j.1558-5646.2010.01029.x) 20482614

[21] Desprez M, McMahon CR, Hindell MA, Harcourt R, Gimenez O. 2013 Known unknowns in an imperfect world: incorporating uncertainty in recruitment estimates using multi-event capture–recapture models. Ecol. Evol. **3**, 4658-4668. (10.1002/ece3.846) 24363895 PMC3867902

[22] Gimenez O et al. 2008 The risk of flawed inference in evolutionary studies when detectability is less than one. Am. Nat. **172**, 441-448. (10.1086/589520) 18657010

[23] Choquet R, Rouan L, Pradel R. 2009 Program E-SURGE: a software application for fitting multievent models. In Modeling demographic processes in marked populations (eds DL Thomson, EG Cooch, MJ Conroy), pp. 845-865. New York, NY: Springer.

[24] Pradel R. 2005 Multievent: an extension of multistate capture-recapture models to uncertain states. Biometrics **61**, 442-447. (10.1111/j.1541-0420.2005.00318.x) 16011690

[25] Kenney RD. 2001 Anomalous 1992 spring and summer right whale (*Eubalaena glacialis*) distributions in the Gulf of Maine. J. Cetacean Res. Manag. (Special Issue) **2**, 209-223.

[26] Whitt AD, Dudzinski K, Laliberté JR. 2013 North Atlantic right whale distribution and seasonal occurrence in nearshore waters off New Jersey, USA, and implications for management. Endanger. Species Res. **20**, 59-69. (10.3354/esr00486)

[27] Winn HE, Price CA, Sorensen PW. 1986 The distributional biology of the right whale (*Eubalaena glacialis*) in the western North Atlantic. Rep. Int. Whaling Commission (Special Issue) **10**, 129-138.

[28] Patrician MR, Biedron IS, Esch HC, Wenzel FW, Cooper LA, Hamilton PK, Glass AH, Baumgartner MF. 2009 Evidence of a North Atlantic right whale calf (*Eubalaena glacialis*) born in northeastern US waters. Mar. Mamm. Sci. **25**, 462-477. (10.1111/j.1748-7692.2008.00261.x)

[29] Kraus SD, Moore KE, Price CA, Crone MJ, Watkins WA, Winn HE, Prescott JH. 1986 The use of photographs to identify individual North Atlantic right whales (*Eubalaena glacialis*). Rep. Int. Whaling Commun. **10**, 145-151.

[30] North Atlantic Right Whale Consortium. 2020 *North Atlantic Right Whale Consortium Entanglements Database 01/20/2020*. Boston, MA: New England Aquarium.

[31] Carpenter B et al. 2017 Stan: a probabilistic programming language. J. Stat. Softw. **76**, 1-32. (10.18637/jss.v076.i01) 36568334 PMC9788645

[32] Stan Development Team. 2021 *Stan modeling language users guide and reference manual. Version 2.28*. See https://mc-stan.org.

[33] R Core Team. 2017 *R: A language and environment for statistical computing*. Vienna, Austria: R Foundation for Statistical Computing. See https://www.r-project.org/.

[34] Stan Development Team. 2020 *RStan: The R interface to Stan.R package version 2.21.2*. See http://mc-stan.org/.

[35] Knowlton AR, Clark JS, Hamilton PK, Kraus SD, Pettis HM, Rolland RM, Schick RS. 2022 Fishing gear entanglement threatens recovery of critically endangered North Atlantic right whales. Conserv. Sci. Practice **4**, e12736. (10.1111/csp2.12736)

[36] Robbins J, Knowlton AR, Landry S. 2015 Apparent survival of North Atlantic right whales after entanglement in fishing gear. Biol. Conserv. **191**, 421-427. (10.1016/j.biocon.2015.07.023)

[37] Harcourt R, van der Hoop J, Kraus S, Carroll EL. 2019 Future directions in *Eubalaena* spp.: comparative research to inform conservation. Front. Mar. Sci. **5**, 530. (10.3389/fmars.2018.00530)

[38] National Marine Fisheries Service. 2019 *North Atlantic Right Whale (Eubalaena glacialis)*. See https://www.fisheries.noaa.gov/species/north-atlantic-right-whale.

[39] Fisheries and Oceans Canada. 2022 *2022 Fisheries management measures to protect North Atlantic Right Whales in Canadian waters*. See https://www.dfo-mpo.gc.ca/fisheries-peches/commercial-commerciale/atl-arc/narw-bnan/2022/right-whale-baleine-noires-0405-eng.html.

[40] Myers HJ, Moore MJ. 2020 Reducing effort in the U.S. American lobster (*Homarus americanus*) fishery to prevent North Atlantic right whale (*Eubalaena glacialis*) entanglements may support higher profits and long-term sustainability. Mar. Policy **118**, 104017. (10.1016/j.marpol.2020.104017)

[41] Reed J, New L, Corkeron P, Harcourt R. 2024 Disentangling the influence of entanglement on recruitment in North Atlantic right whales. Figshare. (10.6084/m9.figshare.c.7095343) PMC10932692 38471549

